# *Roseburia intestinalis*: A possible target for vascular calcification

**DOI:** 10.1016/j.heliyon.2024.e39865

**Published:** 2024-10-26

**Authors:** Xinyun Pan, Yunjian Song, Yapeng Liang, Guoquan Feng, Zhongqun Wang

**Affiliations:** a Department of Cardiology, Affiliated Hospital of Jiangsu University, Zhenjiang, 212001, China; b Institue of Cardiovascular Diseases, Jiangsu University, Zhenjiang, 21200, China; c Department of Emergency, Affiliated Hospital of Jiangsu University, Zhenjiang, 212001, China; d Department of Imaging, Affiliated Hospital of Jiangsu University, Zhenjiang, 212001, China

**Keywords:** Gut microbiome, Vascular calcification, *Roseburia intestinalis*, Butyrate, HDACs, Targeted therapy

## Abstract

With the advancement of metagenomics and metabolomics techniques, the crucial role of the gut microbiome in intestinal, cardiovascular, and metabolic disorders has been extensively explored. Vascular calcification (VC) is common in atherosclerosis, hypertension, diabetes mellitus, and chronic kidney disease. Moreover, it is a significant cause of cardiovascular diseases and mortality. *Roseburia intestinalis*, as a promising candidate for the next generation of probiotics, plays a substantial role in inhibiting the systemic inflammatory response and holds great potential in the treatment of intestinal diseases, cardiovascular diseases, and metabolic disorders. Its primary metabolite, butyrate, acts on specific receptors (GPR43, GPR41, GPR109a). It enters cells via transporters (MCT1, SMCT1), affecting gene expression through HDACs, PPARγ and Nrf2, promoting energy metabolism and changing the concentration of other metabolites (including AGEs, LPS, BHB) in the circulation to affect the body's life activities. In this paper, we focus on the possible mechanism of the primary metabolite butyrate of *Roseburia intestinalis* in inhibiting VC, which may become a potential therapeutic target for the treatment of VC and the ways to enhance its effect.

**Table:** 

Abbreviations	
AGEs	Advanced glycation end products
AS	Atherosclerosis
ALP	Alkaline phosphatase
BMP2	Bone morphogenetic protein 2
ECs	Endothelial cells
FMT	Fecal microbiota transplantation
GLP-1	Glucagon-like peptide 1
HDACs	Histone deacetylases
IBD	Inflammatory bowel disease
ICAM-1	Intercellular adhesion molecule-1
IL	Interleukin
LPS	Lipopolysaccharide
MED	Mediterranean diet
NF-κB	Nuclear factor-kappa B
Nrf2	Nuclear factor erythroid 2-related factor 2
PI3K	Phosphatidylinositol 3 kinase
AKT	The protein kinase B
PPARγ	Peroxisome proliferator-activated receptor-γ
R. intestinalis	Roseburia intestinalis
RAGE	The receptor for advanced glycation end products
ROS	Reactive oxygen species
Runx2	Runt-related transcription factor 2
TNF-α	Tumor necrosis factor-α
Treg cells	Regulatory T cells
VC	Vascular calcification
VCAM-1	Vascular cell adhesion molecule-1
VSMCs	Vascular smooth muscle cells

## 1 Introduction

Vascular calcification has been observed to be common in atherosclerosis, hypertension, diabetes mellitus and chronic kidney disease [[1], [2], [3], [4]]. Vascular calcification is a common pathological phenotype, defined as mineral deposition in the form of calcium phosphate complexes in blood vessels. Vascular calcification has recently been considered an active and highly adjustable process [1]. Vascular calcification is a significant risk factor for major adverse cardiovascular events (MACEs) and is considered to be a crucial cause of cardiovascular disease and mortality [5,6]. Increased production of inflammatory cytokines, heightened oxidative stress, endothelial dysfunction, abnormal glucose and lipid metabolism, and disorders of calcium and phosphorus metabolism can lead to vascular calcification. Endothelial cells (ECs), macrophages, and vascular smooth muscle cells (VSMCs) play an essential role in this process [[7], [8], [9]]. Increased expression of bone morphogenetic protein 2 (BMP2), runt-related transcription factor 2 (Runx2), and osteogenic marker genes, including osteocalcin (OC), collagen IA1 (ColIA1), and osteopontin (OPN), as well as increased alkaline phosphatase (ALP) activity play a crucial role in the transformation of vascular smooth muscle cells (VSMCs) from contractile to osteogenic and promote vascular calcification [[10], [11], [12]]. However, an effective drug for treating vascular calcification in clinical practice still needs to be developed. It is of great clinical significance to explore its possible prevention and treatment.

With the advancement of metagenomics and metabolomics technology, people are increasingly cognizant that the gut microbiome plays a crucial role in intestinal diseases, cardiovascular diseases (CVDs), and metabolic diseases (including obesity, type 2 diabetes mellitus, metabolic syndrome, *etc*.) [[13], [14], [15], [16]]. The composition and function of the gut microbiome are influenced by nearly all major risk factors for vascular calcification, such as ageing, obesity, sedentary lifestyles, and unreasonable diets [17,18]. The FAO/WHO defined probiotics as ‘live microorganisms which, when administered in sufficient amounts, confer a health benefit on the host.’ The next generation of probiotics, including *Roseburia* spp., hold excellent prospects in the future treatment of intestinal diseases, cardiovascular diseases (CVDs), and metabolic diseases [19]. Among them, we detect that *Roseburia intestinalis* may have the potential to treat vascular calcification because it has been demonstrated to produce the metabolite butyrate, which can inhibit inflammatory response and oxidative stress and improve vascular endothelial function [[20], [21], [22]]. Therefore, in this paper, we summarize the possible mechanism by which the primary metabolite butyrate of *Roseburia intestinalis* inhibits vascular VC. This may become a therapeutic target for the treatment of VC and provide insights into ways to enhance the effect of *Roseburia intestinalis*.

## 2 *R. intestinalis* and its metabolite butyrate

*Roseburia intestinalis* (*R. intestinalis*) is an anaerobic, Gram-positive, butyrate-producing bacteria, originally isolated from faeces [23]. *R. intestinalis* belongs to the phylum Firmicutes and is colonized in the mucin layer of the cecum and colon mucosa [24,25]. *R. intestinalis* can generate butyrate, lactic acid, carbon dioxide and hydrogen by fermenting carbohydrates (including glucose, arabinose, cellobiose, maltose, fructose, raffinose, sucrose, xylose, xylan and starch) and acetic acid. Additionally, arylamidase activity is not detected when using the Rapid ID-32 A system [23]. Therefore, diet-related substrates are crucial for the production of butyrate. Kim et al. have demonstrated that, compared with other butyrate-producing bacteria, live *R. intestinalis* produces more butyrate, digests more complex prebiotic polysaccharide structures, and can significantly increase the gene expression levels of tight junction proteins (ZO-1, occludin, claudin 3) and improve the intestinal mucosal barrier [26]. Moreover, studies have shown that it is harmless and resistant to gastric acid, enabling it to reach the intestine and play a role [[24], [25], [26]]. Compared with other probiotics, *R. intestinalis* can form a higher proportion of biofilms, thereby inhibiting the colonization of pathogens on the mucosa and improving the gut microbiome [24]. Lastly, establishing Ri 7_2 (an aptamer library) makes it possible to detect *R. intestinalis* quickly and economically, which is conducive to its clinical application as a probiotic [27].

The abundance of *R. intestinalis* significantly decreases in a variety of diseases, including diabetes mellitus [28], metabolic syndrome [29], AS [30], colorectal cancer, inflammatory bowel disease (IBD) [22,31,32], nonalcoholic fatty liver disease [33], alcoholic fatty liver [34], *etc. R. intestinalis* plays an essential role in inhibiting the inflammatory response and in the treatment of intestinal diseases, cardiovascular diseases, and metabolic diseases. Its metabolite butyrate promotes the anti-inflammatory properties of colonic macrophages by acting on the GPR109a. It also induces Regulatory T cells (Treg cells) and promotes the expression of the anti-inflammatory cytokines thymic stromal lymphopoietin (TSLP), transforming growth factor-β (TGF-β) and IL-10, thereby inhibiting colonic inflammation and carcinogenesis [22,35]. *R. intestinalis* plays a role in the treatment of atherosclerosis by producing butyrate, reducing endotoxemia, improving intestinal barrier function, and reducing inflammatory cytokines in plasma and aorta [30]. *R. intestinalis* plays a role in the treatment of atherosclerosis by producing butyrate. This leads to a reduction in endotoxemia, an improvement in intestinal barrier function, and a decrease in inflammatory cytokines in plasma and the aorta [36]. Therefore, *R. intestinalis*, by inhibiting the inflammatory response, may serve as a potential therapeutic target for vascular calcification through its metabolite butyrate.

## 3 The role of butyrate in the course of vascular calcification

Using Spearman's correlation analysis, Yan et al. found that butyrate levels in plasma and faecal samples negatively correlate with calcification scores [20]. In patients with chronic diseases, compared with the high calcification score group, the butyrate-producing bacteria in the low calcification score group was significantly more abundant, indicating that butyrate may be closely related to vascular calcification [37]. Interestingly, Kasahara et al. proved that *R. intestinalis* affects gene expression by producing butyrate, reduces the inflammatory response, and reduces atherosclerosis [30]. So, we strongly speculate that *R. intestinalis* may inhibit the inflammatory response and reduce the occurrence of vascular calcification, which is considered to be the end stage of atherosclerosis [1,3]. Butyrate is a metabolite produced by anaerobic fermentation of dietary polysaccharides, including starch and non-starch polysaccharides (dietary fiber) in the colon [38]. It enters the cells through Na + -coupled transporter SLC5A8 and the H + -coupled transporter SLC16A1 on the intestinal epithelium. It is oxidized to form acetyl CoA, which enters the tricarboxylic acid cycle to produce ATP, the primary energy source of colonic epithelial cells [38,39]. At the same time, butyrate can also enter the circulation from the intestine through the liver and portal vein system, and butyrate plays an essential role in systemic energy homeostasis because the transporters SLC5A8 and SLC16A1 are widely expressed throughout the body [[38], [39], [40]]. Butyrate plays a physiological role by acting on specific receptors (GPR43/FFAR2, GPR41/FFAR3 and GPR109a/HCAR2) and entering cells through transporters (MCT1/SLC16A1 and SMCT1/SLC5A8) and these receptors are widely expressed in peripheral tissues [39]. Butyrate may affect gene expression, signal transduction pathways, and the concentration of other metabolites in the body, thereby alleviating vascular calcification [15,21,41,42].

### 3.1 Specific receptors

GPR43, GPR41 and GPR109a are widely expressed in peripheral tissues. We mainly discussed the role of butyrate in the course of vascular calcification by acting on these receptors, including in intestinal epithelial cells and immune cells [39].

#### 3.1.1 GPR43 and GPR41

**Fig. 1:**
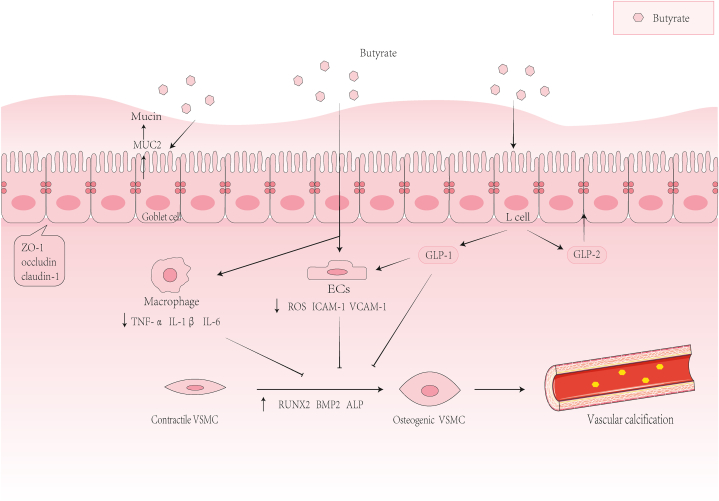
Butyrate may inhibit vascular calcification. Butyrate may inhibit the transformation of VSMCs from contractile to osteogenic and vascular calcification by inhibiting macrophage inflammatory response and endothelial dysfunction. Butyrate improves the intestinal barrier by promoting mucus secretion and improving tight junctions to inhibit LPS to promote VC.

By activating GPR43 and GPR41 on L-cells, butyrate can increase the concentration of glucagon-like peptide 1 (GLP-1). This may improve the endothelial function, reduce the inflammatory response, and may have a protective effect on vascular calcification [[43], [44], [45], [46]]. Studies have shown that GLP-1R is expressed in vascular endothelial and smooth muscle cells and protects CVDs [45], [47]. Johanna et al. proved that GLP-1 inhibits an inflammatory cascade involving Nuclear factor-kappa B (NF-κB) and downregulates mRNA encoding vascular adhesion molecules such as vascular cell adhesion molecule-1 (VCAM-1), intercellular adhesion molecule-1 (ICAM-1). It also improves vascular endothelial function by acting on GLP-1R in vascular endothelial cells [48] (Fig. 1). Zhan et al. demonstrated that GLP-1 may reduce the protein expression of osteoblast differentiation markers ALP, OC, and Runx2 by inhibiting the phosphatidylinositol 3 kinase (PI3K)/The protein kinase B (AKT)/mTOR/S6K1 pathway. They weakened osteoblast differentiation and vascular calcification of human VSMCs [49] (Fig. 1).

#### 3.1.2 GPR109a

Butyrate is the only short-chain fatty acid that acts on GPR109a. Butyrate can promote the differentiation of Treg cells and IL-10-producing T cells induced by colonic macrophages through the GPR109a signaling pathway [22]. In vitro, *R. intestinalis* has been shown to induce the differentiation of Treg cells in human peripheral blood mononuclear cells [35]. Studies have shown that high glucose promotes the phenotypic transformation of macrophages from the anti-inflammatory M2 phenotype (producing anti-inflammatory factors IL-10 and TGF-β) to the pro-inflammatory M1 phenotype (producing pro-inflammatory factors IL-β, IL-6, TNF-α), thereby aggravating vascular calcification [9,50]. Butyrate can promote the formation of Treg cells outside the thymus, thereby promoting the conversion of the M1 phenotype to the M2 phenotype, inhibiting pro-inflammatory response. It may play a role in inhibiting vascular calcification [51,52].

### 3.2 Gene expression

Previous research has shown that butyrate may alleviate inflammatory response and endothelial dysfunction by affecting gene expression of macrophages, VSMCs and ECs through HDACs, PPARγ, Nrf2, *etc*. [42,53]. We summarized butyrate's role in vascular calcification through its influence on gene expression.

#### 3.2.1 HDCAs

Butyrate can inhibit the NF-κB signaling pathway and histone deacetylases (HDACs) in monocytes and macrophages. This promotes the production of anti-inflammatory factor IL-10 and inhibits the production of LPS and pro-inflammatory factors such as tumour necrosis factor-α (TNF-α), IL-6, IL-1β, and NO. Thus, it reduces the inflammatory response, inhibit the osteogenic differentiation of vascular smooth muscle, and inhibits vascular calcification [42] [54], [55], [56], (Fig. 1). Arpaia et al. have shown that HDAC inhibitor vorinostat butyrate inhibits IL-1β-induced expression of MMPs and iNOS by inhibiting the phosphorylation of NF-κB and p38 and ERK1/2. This reduce vascular calcification [57]. In order to explore the effect of butyrate on vascular calcification through HDACs, we summarized the possible effects of different types of HDACs on vascular calcification (Table 1). In contrast, a study has shown that butyrate significantly activats NF-κB signaling and inhibits HDAC2, HDAC3, HDAC8 and HDAC11 in VSMCs and promotes vascular calcification [58]. However, the effect on NF-κB as proven in this article is contrary to that in most other articles. In response to this inconsistency, we hypothesize that *R. intestinalis* may produce a more appropriate concentration of butyrate to affect vascular calcification. Animal experiments have demonstrated that the concentration of butyrate around the artery is not very high. As a result, direct effect of butyrate on VSMCs may not be dominant, and the direct effect of butyrate on cells may not be entirely accurate [30]. The colonization experiment of *R. intestinalis* in intestine of mice is indeed a promising research direction. In addition to investigating the direct effect of butyrate on VSMCs, it is intriguing that butyrate can also have an impact on ECs [59]. In HG-treated ECs, butyrate inhibited HDAC activity. This led to a reduction in reactive oxygen species (ROS) production and mRNA expression of VCAM-1 and ICAM-1. By reducing oxidative stress and inflammation, butyrate alleviated endothelial dysfunction. This is believed to play a role in alleviating vascular calcification [3], [59].

**Table 1:** The possible effects of different types of HDACs on vascular calcification.

Types	Relationship with vascular calcification	Mechanism	References
Ⅰ			
HDAC1	inhibition	Down-regulation of Runx2	[60]
HDAC2	inhibition	Promotion of vascular smooth muscle cells to contractile VSMC	[61]
HDAC3	promotion	Promotion of vascular smooth muscle cell proliferation and migration	[62]
HDAC8	inhibition	Associating with smooth muscle α-actin (α-SMA) and essential for smooth muscle cell contractility	[63]
Ⅱa			
HDAC4	promotion	Binding the adaptor protein ENIGMA (Pdlim7) in the cytoplasm	[64]
HDAC5	inhibition	Maybe through down-regulation of Runx2, BMP2 and up-regulation of OPG	[65]
HDAC7	inhibition	Down-regulation of Runx2, inhibiting angiogenesis and osteoblast differentiation	[66]
HDAC9	promotion	Up-regulation of Runx2, changing VSMC phenotype, decreasing contractility and increasing proliferation and activating NF-κB signaling pathway, therefore, increasing the release of inflammatory factors	[67] [68],
Ⅱb			
HDAC6	promotion	Mediating endothelial dysfunction and atherosclerosis	[3] [69],
HDAC10	promotion	Activating proliferation and autophagy of VSMC, which is enhanced by regulating PCNA and EZH2	[70] [71],
Ⅲ			
SIRT1	inhibition	Maybe inhibiting the formation of osteogenic VSMC by inhibiting the expression of p21 and Runx2	[72]
SIRT2	inhibition	Inhibition of p66^shc^ to reduce the production of mROS, inhibiting ageing-induced vascular remodelling.	[73]
SIRT3	inhibition	Increasing the expression of antioxidant enzymes, including SOD1 and SOD2, inhibiting oxidative stress and formation of osteogenic VSMC	[74]
SIRT4	inhibition	Improving endothelial dysfunction	[75]
SIRT5	inhibition	Improving mitochondrial dysfunction of endothelial progenitor cells	[76]
SIRT6	inhibition	Inhibiting the formation of osteogenic VSMC by regulating Runx2	[77]
SIRT7	inhibition	Reducing the inflammatory response of endothelial cells	[78]

#### 3.2.2 PPAR

Activation of Peroxisome proliferator-activated receptor-γ (PPARγ) has been discovered to play an essential role in cardiovascular and metabolic diseases by inhibiting inflammatory response and increasing insulin sensitivity [79]. Butyrate is considered to stimulate PPAR-γ signaling. This has several important consequences. Firstly, it inhibits the synthesis of inducible nitric oxide synthase (iNOS) in the intestine. As a result, there is an increased expression of nitrate levels. Secondly, it induces the energy metabolism of colon epithelial cells to develop in the direction of β-oxidation. This helps maintain hypoxia in colon cells and promotes the growth of anaerobic bacteria. By regulating intestinal flora imbalance in these ways, butyrate is favorable to health [53]. In ECs, PPARγ plays a crucial role. It inhibits age-related vascular endothelial dysfunction by reducing oxidative stress and minimizing the inflammatory response [80]. In VSMCs, PPARγ exerts a significant inhibitory effect on vascular calcification. It does so by activating secreted frizzled-related protein-2 (sFRP2), which in turn inhibits the chondrogenic Wnt5 pathway [81]. In macrophages, the activation of PPARγ promotes macrophage phenotype conversion by promoting glutamine metabolism and inhibits inflammatory response [82]. However, the effect of butyrate on the PPARγ signaling pathway in different cells still requires more in-depth study to fully understand its function.

#### 3.2.3 Nrf2

Activation of nuclear factor erythroid 2-related factor 2 (Nrf2) is of crucial importance in inhibiting oxidative stress and inflammation-induced chronic diseases [83]. Overall, studies have shown that the absence of Nrf2 may play an essential role in the formation of atherosclerotic plaque calcification [84]. In ECs, butyrate exerts a protective effect on vascular calcification. This is achieved through activating Nrf2 transcription. By doing so, it reduces ROS production and the mRNA expression of VCAM-1, ICAM-1, reducing oxidative stress and inflammatory response, improving endothelial dysfunction, reducing endothelial cell permeability. As a result, VSMCs have less contact with pro-inflammatory factors [3] [59], (Fig. 1). In VSMCs, the activation of Nrf2 inhibits vascular calcification by inhibiting the activity of Runx2, as well as reducing oxidative stress and apoptosis of VSMCs [85], [86]. As previously noted, butyrate may inhibit vascular calcification by acting on both endothelial cells and smooth muscle cells [84] [85], [86].

#### 3.2.4 PI3K/AKT

Butyrate has been demonstrated to inhibit the proliferation, apoptosis and migration of VSMCs by inhibiting HDACs and PI3K/AKT pathway. This leads to cell enlargement and a change in cell shape from a slender bipolar form to a circular diffusion phenotype, which may play a protective role in vascular calcification [71] [87], [88]. Meanwhile, studies have provided evidence that apoptosis of smooth muscle cells can contribute to vascular calcification. This occurs through the production of apoptotic bodies, which in turn promotes the accumulation of calcium within these bodies and also stimulates autophagy [71], [85]. In addition, butyrate may inhibit the expression of Runx2 and vascular calcification by inhibiting the PI3K/AKT pathway [12] [49], [88], [89].

### 3.3 Other metabolites

Although butyrate can enter the circulation and potentially regulate cell function, its concentration within the circulation and its specific effects on VSMCs and other cells require further investigation [39]. Therefore, in an effort to better understand the role of butyrate in vascular calcification, we have also explored how butyrate might further affect this process by influencing other metabolites. This includes AGEs, LPS, and BHB.

#### 3.3.1 AGEs

**Fig. 2:**
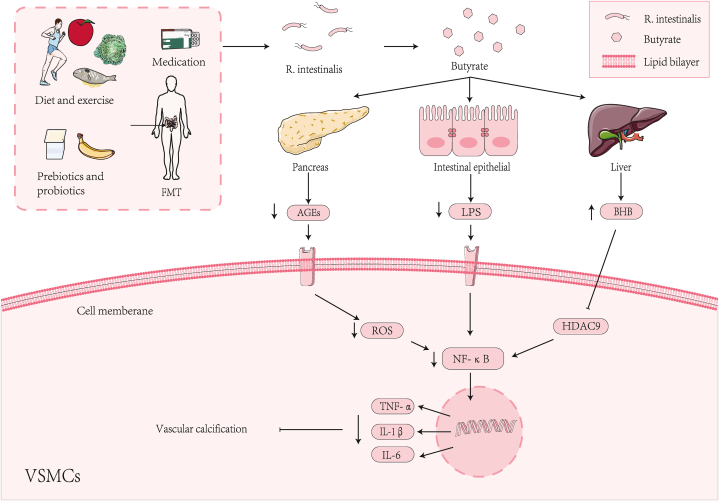
Some factors are conducive to increasing the abundance of R.intestinalis to increase butyrate. Diet, exercise, prebiotics, probiotics, medication and FMT may increase the abundance of *R. intestinalis* to increase butyrate. Butyrate inhibits a series of inflammatory reactions mediated by the NF-κB signaling pathway. Then, it inhibits vascular calcification by increasing insulin and inhibiting glucagon secretion to inhibit the content of ACEs, improving the intestinal barrier to inhibit the content of LPS, and promoting the ketogenic effect to increase the content of BHB in the blood.

DM is indeed one of the most common endocrine diseases. Type 2 diabetes mellitus (T2DM) is characterized by insulin resistance and pancreatic β-cell dysfunction, which leads to persistent hyperglycemia and a high risk of cardiovascular complications [90]. Advanced glycation end products (AGEs) are significantly increased in diabetic patients and are closely related to diabetic vascular calcification [91], [92]. AGEs and the receptor for advanced glycation end products (RAGE) constitute the AGEs/RAGE signaling pathway, which activates MAPKs and NF-κB signaling pathways by activating the production of ROS in cells, and increases the expression of BMP-2, Runx2 and ALP activity, participating in the progression of vascular calcification [3] [93], (Fig. 2). The AGEs/RAGE signaling pathway has a significant impact on promoting diabetic vascular calcification. When this pathway is activated, it can aggravate oxidative stress in several ways, including activating NADPH oxidase-1 (Nox-1) and reducing the expression of Cu/Zn superoxide dismutase (SOD-1) [91]. Interestingly, butyrate shows potential in alleviating AGEs-induced vascular calcification by relieving diabetes, reducing blood glucose and inhibiting insulin resistance [93], [94]. In terms of mechanism, butyrate plays a significant role by mainly promoting the secretion of GLP-1 and PYY by acting on GPR41 and GPR43 of enteroendocrine L-cells [15], [95]. GLP-1 and PYY play crucial roles in regulating blood glucose through multiple mechanisms, including regulating appetite and satiety, increasing insulin and inhibiting glucagon secretion [95], [96]. Butyrate exerts a protective effect on pancreatic beta cells. By inhibiting the activity of histone deacetylases (HDACs) and the NF-κB signaling pathway, butyrate reduces the expression of the inflammatory factor IL-1β. This is significant as high levels of IL-1β can cause inflammation and damage to pancreatic beta cells [97]. What is more, butyrate shows significant potential in addressing insulin resistance and persistent hyperglycemia by acting on multiple tissues such as skeletal muscle, liver, and adipose tissue [98] [99], [100].

#### 3.3.2 LPS

Lipopolysaccharide (LPS), which constitutes the main component of the outer membrane of Gram-negative bacteria, can translocate into the blood microcirculation when an imbalance in the intestinal flora gives rise to increased intestinal permeability. Subsequently, LPS binds to toll-like receptor 4 (TLR4) on the target tissue, thereby inducing endotoxemia and eliciting a systemic inflammatory response [101] [102], [103]. LPS can induce the MAPKs and NF-κB pathways in aortic smooth muscle cells. This stimulation leads the cells to produce a variety of pro-inflammatory cytokines, such as TNF-α, IL-1β and IL-6 (Fig. 2). Simultaneously, LPS may promote vascular calcification by inducing endoplasmic reticulum stress in VSMCs [104], [105]. Among them, TNF-α is the main mediator mediating the systemic inflammatory cascade induced by LPS, which promotes the transdifferentiation and calcification of VSMCs mainly by activating the NF-κB pathway [106], [107]. Since LPS has such an adverse effect on vascular calcification, reducing it is essential. Butyrate may protect the intestinal barrier by increasing mucus secretion and enhancing tight junctions. Subsequently, by preventing LPS from entering the circulation, it helps to reduce vascular calcification [105], [108]. Butyrate may promote intestinal epithelial goblet cells to secrete mucus and protect the intestinal barrier by up-regulating the expression of MUC2 [108] [109], (Fig. 1). Butyrate can also enhance the intestinal barrier by accelerating the assembly of tight junction proteins zonula occludens-1 (ZO-1) and occludin through increasing the activity of AMPK [110]. What is more, butyrate can also promote the secretion of GLP-2. This, in turn, increases the expression of ZO-1 and occludin and improves the intestinal mucosal barrier. Consequently, it prevents lipopolysaccharide (LPS) from entering the circulation [111] [112], (Fig. 1).

#### 3.3.3 BHB

Butyrate can promote the ketogenic effect in the liver by improving the catalytic activity of 3-hydroxy-3-methylglutaryl-CoA synthase 2 (HMGCS2) and then increasing the concentration of β-hydroxybutyric acid (BHB) in the circulation [113] (Fig. 2). BHB exerts anti-inflammatory effects by reducing the production of IL-1β and IL-18 mediated by the nod-like receptor family pyrin domain containing 3 (NLRP3) inflammasome in human monocytes [114]. Among them, activation of the NLRP3 inflammasome promotes vascular calcification [115]. BHB has been shown to inhibit vascular calcification by down-regulating the HDAC9-dependent NF-κB signaling pathway under high phosphorus induction [68]. What is more, HDAC9 has also been proven to promote the osteogenic differentiation of VSMCs and the formation of VC by up-regulating NF-κB and Runx2 [67], [68]. In the high phosphate-induced model, BHB can also inhibit vascular calcification by enhancing autophagy. It does so by promoting the fusion of autophagosomes and lysosomes to form autophagolysosomes [116].

### 3.4 Mitochondrial function

Mitochondrial dysfunction is an important pathological feature of vascular ageing, which plays a vital role in the pathogenesis of vascular diseases associated with ageing and can lead to AS [117], [118]. Mitochondrial dysfunction is characterized by mtDNA damage, decreased ATP synthesis, and increased oxidative stress, ultimately leading to VC [119]. Studies have shown that butyrate induces mitophagy by activating AMPK, thereby reducing ROS production and alleviating oxidative stress [120], [121]. Butyrate alleviates mitochondrial dysfunction by increasing the mRNA expression levels of mitochondrial function-related genes TFAM, NRF-1 and PGC-1α [120]. Butyrate may play an essential role in inhibiting oxidative stress by up-regulating the expression of uncoupling protein 2 (UCP2), an essential gene related to PPARγ-mediated uncoupling, and promoting the synthesis of ATP [40], [122]. Butyrate promotes the uptake of butyrate and fatty acid oxidation by promoting the expression of MCT1 and ACSM3, thereby promoting ATP synthesis [40]. Therefore, butyrate may play a protective role in VC by improving mitochondrial dysfunction.

## 4 Targeted *R. intestinalis* to treat and improve vascular calcification

*R. intestinalis* has been shown to play an essential role in the treatment of AS, intestinal inflammatory diseases and metabolic syndrome by producing butyrate and improving the intestinal barrier [22] [29], [30]. In addition, diet, exercise, prebiotics, probiotics, medication and FMT also play an important role in increasing the abundance of *R. intestinalis* in the body. This may help to treat and improve VC (Table 2) (Fig. 2).

**Table 2:** Diet, exercise, prebiotics, probiotics, drugs, and FMT may help increase the abundance of *R. intestinalis* in the body and promote human health.

Types	Subjecs	Time	Outcomes	Diseases	References
Diet					
Omega-3 rich diets	45-year-old male	2W	*Roseburia* spp.↑	Chronic diseases	[123]
MED	Human	4Wand 8W	*R. intestinalis*↑	Obesity	[124]
PPT	Pre-diabetic individuals	6M	*R. intestinalis*↑	Diabetes mellitus	[125]
Prebiotics					
diet with HPP	ApoE−/−Mice	18W	*R. intestinalis*↑	Atherosclerosis	[30]
CCPP	Mice	6W	*R. intestinalis*↑	Diabetes mellitus	[126]
arabinoxylans	Mice	4W	*Roseburia* spp.↑	obesity	[127]
Medication					
metformin	human	2W	*Roseburia* spp.↑	Diabetes mellitus	[128]
vitamin D	CD patients	1W	*Roseburia* spp.↑	Crohn's disease	[129]

HPP: High content of plant polysaccharides.

CCPP: Cyclocarya paliurus polysaccharides.

### 4.1 Diet and exercise

Studies have shown that a healthy diet and moderate exercise may play an essential role in improving human health by changing the gut microbiome and its metabolites [130], [131]. A study has shown that a short-term animal-based diet leads to a decrease in the abundance of *Roseburia* spp. This decrease is unrelated to individual differences in microbial gene expression [132]. The Mediterranean diet (MED), characterized by a high intake of healthy food groups such as vegetables, fruits, and fish, reduces circulating LPS levels. It does so by reducing the concentration of circulating 3-hydroxy fatty acids (3-OH FAs), thereby reducing inflammatory responses. Additionally, it can treat obesity by increasing the abundance of *Roseburia* spp. [123], [133]. Interestingly, in a recent clinical study of dietary interventions in pre-diabetes, Shoer et al. proved that a personalized postprandial glucose-targeting diet (PPT) had a greater effect on microbiome and metabolites than MED, including the relative abundance of 19 gut microbiomes. At the same time, MED only affected four. Therefore, dietary intervention based on microbiome features is more conducive to affecting the composition of the gut microbiome, which may help to increase the abundance of *R. intestinalis* and butyrate in a more individualized manner [124].

Some scholars have shown that after gut microbiome transplantation from exercise-trained mice, the recipient mice have a higher proportion of butyrate-producing bacteria and an increased butyrate production [134]. In subsequent human studies, they verified that exercise can promote the production of butyrate-producing bacteria, including *Roseburia* spp. and butyrate [135]. In addition, they also found that thin subjects changed more significantly than fat subjects. However, this phenomenon needs further exploration [135]. Liu et al. speculated that different people have different responses to exercise to improve glucose metabolism and insulin sensitivity. This may be because different individuals have different gut microbiome community structures [136]. Therefore, gut microbiome community structure may be a marker to predict the efficacy of exercise intervention in changing the gut microbiome [136]. Interestingly, butyrate has also been proven to improve insulin resistance by synthesizing SCFAs, enhancing its binding to GPR43, and increasing autophagy in skeletal muscle cells [98].

### 4.2 Prebiotics, probiotics and synbiotics

Both prebiotics and probiotics play an essential role in regulating the intestinal microbiome and improving the intestinal barrier [137] [138], [139]. Prebiotics and probiotics have been reported to hold great promise in the treatment of obesity, diabetes, IBD and other metabolic diseases [140], [141]. The panel synbiotic as 'a mixture comprising living microorganisms and substrate(s) selectively utilized by hosts that confers a health benefit on the host.' This definition helps to elucidate the cooperation of prebiotics and probiotics in promoting health [142]. Studies have shown that prebiotic polysaccharides, including excipients, arabinoxylan, and pullulan rather than inulin, can be utilized by *R. intestinalis* to promote the production of butyrate, thereby enhancing its function [26]. β-mannan has been shown to protect the mucus barrier and R. intestinals has been proved to be its main user. As a result, β-mannan play a role in promote the increase of *R. intestinalis* and the production of butyrate [143]. Prebiotics may also increase the level of butyrate by stimulating mucus secretion and stimulating the release of mucosal *R. intestinalis* into the lumen [25]. Cyclocarya paliurus polysaccharides (CCPP) as a carbon source can improve glucose tolerance and insulin resistance by increasing the abundance of R. intestinnals and butyrate [126]. In addition, R. intestinnals and Faecalibacterium prausnitzii can promote the utilization of β-mannan and increase the concentration of metabolite butyrate through a collaborative way known as cross-feeding [144]. Probiotics play an essential role in restoring the dysregulated microbiome and regulating the microecological balance of the gut microbiome through cross-feeding [145], [146]. By degrading undigestible prebiotics and producing acetic acid, bifidobacterium provides raw materials for *R. intestinalis* and promotes the production of butyrate [146]. Considering the safety of probiotic use, Prebiotics-Encapsulated Probiotic Spores (spores-dex) have been produced by researchers to increase the concentration of SCFAs produced in the intestine, and at the same time, they also have been proved to increase the abundance of the gut microbiome, including *Roseburia* spp. [147]. Therefore, synbiotics treatment is more conducive to the role of *R. intestinalis*.

### 4.3 Medication

In recent years, the relationship between the gut microbiome and non-antibiotic drugs has indeed received growing attention, and the effects and side effects of many drugs may be closely related to gut microbiome [128] [148], [149]. In a randomized, double-blind study of newly diagnosed T2DM individuals, metformin has been shown to alter the composition and function of the gut microbiome to reduce blood glucose [150]. In a large cohort study of 2173 healthy people or patients with common chronic diseases, the researchers found that the combination of drugs, such as diuretics combined with aspirin, ACE inhibitors or β-blockers, was more conducive to increasing the abundance of *Roseburia* spp. and improving health. Metformin and statin doses have been proven to correlate positively with the abundance of *R. intestinalis* [151]. Butyrate production seems closely related to the utilization rate of iron, therefore, supplementing an appropriate amount of iron to maintain a suitable iron level in the intestine helps to promote butyrate production by *R. intestinalis* [152].

### 4.4 FMT

Faecal microbiota transplantation (FMT) is a revolutionary approach that involves transplanting the faecal microbiota from healthy individuals into patients. Moreover, FMT may also play a significant role in treating IBD, obesity, and DM [153]. FMT can restore the gut microbiome after antibiotic use, which may benefit the growth of symbiotic members of the microbiome [137]. After receiving thin donor FMT, the gut microbiome diversity of male subjects with untreated metabolic syndrome increases significantly, and the abundance of *R. intestinalis* increases by 2.5 times, thereby improving insulin resistance [154]. Further more, research on oral FMT is currently underway, and it has been shown that therapeutic prospects in patients with ulcerative colitis show advantages in terms of accessibility and tolerance [155]. However, FMT indeed faces many challenges, such as the risk of infectious disease transmission and the uncertainty of the receptor immune system's response to the donor flora [156], [157]. In conclusion, whether FMT can treat vascular calcification-related cardiovascular diseases may require more experimental verification.

## 5 Conclusion

In general, *R. intestinalis* mainly inhibits the systemic inflammatory response through the metabolite butyrate and plays a vital role in the treatment of intestinal, cardiovascular, and metabolic diseases. The direct effect of butyrate on VSMCs still requires clarification, and more laboratory experiments and clinical trials are needed to verify its efficacy. However, it is found that butyrate may inhibit VC by reducing macrophage inflammatory response and endothelial dysfunction and changing the concentration of other metabolites in the circulation, such as reducing AGEs and LPS and increasing BHB. Additionally, it is observed that diet, exercise, prebiotics, probiotics, medication, and FMT can significantly increase the abundance of intestinal *R. intestinalis* and play an essential role in treating several diseases. Currently, the effect of *R. intestinalis* colonization in mice or humans on VC is still limited. Therefore, more experiments are needed to verify the role of *R. intestinalis* in the treatment of VC, such as the colonization experiment of *R. intestinalis* in the intestine of mice or humans and the effects of different diet, exercise, prebiotics, probiotics, medication, and FMT on the content of *R. intestinalis* and its production of butyrate in vivo. These studies may further prove that whether *R. intestinalis* can become one of the effective methods for the treatment of VC.

## CRediT authorship contribution statement

**Xinyun Pan:** Writing – review & editing, Writing – original draft. **Yunjian Song:** Writing – review & editing. **Yapeng Liang:** Writing – review & editing. **Guoquan Feng:** Writing – review & editing. **Zhongqun Wang:** Writing – review & editing.

## Informed consent statement

Not applicable.

## Institutional review board statement

Not applicable.

## Data availability

Not applicable.

## Funding

This work was supported by grants from the 10.13039/501100001809National Natural Science Foundation of China (82370457, 82070455), Key Research & Developement Plan of 10.13039/501100002949Jiangsu Province (BE2022780), the Project from Social development of Zhenjiang (FZ2022088).

## Declaration of Competing Interest

The authors declare that they have no known competing financial interests or personal relationships that could have appeared to influence the work reported in this paper.
